# Monitoring Student Activities with Smartwatches: On the Academic Performance Enhancement

**DOI:** 10.3390/s19071605

**Published:** 2019-04-03

**Authors:** Oscar Herrera-Alcántara, Ari Yair Barrera-Animas, Miguel González-Mendoza, Félix Castro-Espinoza

**Affiliations:** 1 Departamento de Sistemas, Universidad Autónoma Metropolitana, Azcapotzalco 02200, Mexico; 2 Centro Universitario UAEM Valle de México, Universidad Autónoma del Estado de México, Atizapán 54500, Mexico; 3 Escuela de Ingeniería y Ciencias, Tecnológico de Monterrey, Atizapán 52926, Mexico; ybarrera@tec.mx (A.Y.B.-A.); mgonza@tec.mx (M.G.-M.); 4 Centro de Investigación en Tecnologías de Información y Sistemas, Universidad Autónoma del Estado de Hidalgo, Mineral de la Reforma 42184, Hidalgo, Mexico; fcastro@uaeh.edu.mx

**Keywords:** human activity recognition, smartwatch sensors, supervised classification

## Abstract

Motivated by the importance of studying the relationship between habits of students and their academic performance, daily activities of undergraduate participants have been tracked with smartwatches and smartphones. Smartwatches collect data together with an Android application that interacts with the users who provide the labeling of their own activities. The tracked activities include eating, running, sleeping, classroom-session, exam, job, homework, transportation, watching TV-Series, and reading. The collected data were stored in a server for activity recognition with supervised machine learning algorithms. The methodology for the concept proof includes the extraction of features with the discrete wavelet transform from gyroscope and accelerometer signals to improve the classification accuracy. The results of activity recognition with Random Forest were satisfactory (86.9%) and support the relationship between smartwatch sensor signals and daily-living activities of students which opens the possibility for developing future experiments with automatic activity-labeling, and so forth to facilitate activity pattern recognition to propose a recommendation system to enhance the academic performance of each student.

## 1. Introduction

Human activity monitoring and recognition have become a relevant research area in the last decades given the applications in medical diagnosis, fitness training, healthy life style, entertainment. and education [1,2,3,4,5,6].

Smartwatches, smartphones, and other mobile devices have become popular and have supported human activity recognition in real world situations, once they allow assistance on daily user activities, in contrast with other complex devices that require sophisticated laboratories and have minimal mobility.

Several works report the use of smartphones and smartwatches in the monitoring of activities. For example, Pernek et al. [7] described how smartphones can be used to capture data from training-resistance to give reliable training feedback; a 1% of miscount rate is achieved when detecting individual resistance training repetitions.

van Dantzig et al. [8] developed a mobile application, named SitCoach, for monitoring physical activity and sedentary behavior to provide timely persuasive messages that suggest active breaks for office employees.

Context detection is studied by Pei et al. by combining positioning technologies and phone-sensors to capture human movements and their data to study human behavior [9].

Wundersitz et al. [10] determined if data of a single wearable tracking device can be used to classify activities related to a sports team.

Duclos et al. [11] proposed an energy-saving function to estimate the total energy consumed by a person. The energy function is implemented in an application that combines smartphone and smartwatch accelerometer data to discriminate human behaviors.

Activities of sitting, standing, walking, running, cycling, stair descent, stair ascent, elevator descent, and elevator ascent were studied by Guiry et al. [12] with smartphones and smartwatches. The dataset was balanced to give all activities an equal representation. Single and multi-sensor approaches were studied with models that classify correctly up to 100% of all instances when differentiating between indoors and outdoors. The protocol considers walking between buildings conducted during daylight hours by using GPS and light sensors.

In addition, for activity recognition, Shoaib et al. [13] used three classifiers to recognize 13 different activities, such as walking, typing, writing, jogging, biking, walking upstairs, walking downstairs, sitting, standing, eating, drinking coffee, giving a talk, and smoking, and they comment about the need for fusing smartwatches and smartphones to recognize such activities with reasonable accuracy. Eating, drinking a cup of coffee, giving a talk, and smoking are considered complex activities.

Students’ behavior and academic performance seem to be more related to intelligent tutoring systems given the evidence of the use of technologies, such as (Electroencephalograph) EEG-headsets, microphones, cameras, among other devices [14].

EEG-headsets have been used to build tutoring systems by detecting a student’s emotions when studying [15]. In particular, Mehmood et al. [16] presented a computer aided education system with the ability to recognize emotional activities that responds to a student’s emotions to improve their learning capabilities. Despite successful applications, a drawback of EEG-devices is that they require special conditions to operate properly, including the use of saline solutions and the right placing of electrodes on the scalp.

Microphones have been used with interactive conversation systems [17] which are often designed for personal computers and, to get better results, is preferably uses in laboratories with low environmental noise which yield low portability.

Kunze et al. [18] showed how eye tracking glasses can be used to count the words when reading and to know the reading habits of users which give a place to prototypes of cognitive activity recognition systems. In addition, eye-tracking glasses and web cameras have been used together for monitoring concentration of learners based on analyzing pupillary response obtained from the eye tracker and eye-blinking patterns obtained from a web camera to develop intelligent tutoring systems [19]. Since eye-tracking devices and web cameras require the procession by software and the use of computers, it leads to use in specialized laboratories.

Devices that combine cameras, microphones, and depth sensors, such as the Microsoft Kinect [20], have been applied in education [21]. Reference [22] presented as an interactive technology that facilitates teaching and learning by supporting kinesthetic pedagogical practices. However, there are technical constraints when using in the classroom: the need for a large classroom space, long calibration time, computer, projector, and compatible software.

Compared with EEG-devices, eye tracking devices, microphones, and cameras for personal computers, smartphones and smartwatches represent an accessible and mobile alternative to develop wearable applications. Even though several researches have reported the use of different technologies in human activity monitoring and intelligent tutoring systems, we have not found enough evidence of smartphones and smartwatches used to track students’ activities and any relationship with academic performance. Few researches have addressed the activity monitoring focused on academic performance using wearables devices. For example, Leitão et al. [23] presented an experimental evaluation of machine learning supervised techniques in human activity recognition where smartphones sensors data are collected with the overall goal of recognizing activities to identify students with attention deficit or hyperactivity problems based on three activities: walking, standing, and sitting.

By assuming that the daily activities of students are associated with their scholar scores, we consider it convenient to know how their habits affect their academic performance. For example, in [24] it is reported that poor sleep quality has a great impact on the grade-point average, but many times it is considered out of the scope of the universities planning. Moreover, poor course-scheduling and an excessive homework load contribute negatively to scholar scores.

We aim to contribute to the study of daily-living activities of students based on the hypothesis that there are relationships between their academic performance and their habits, and between activities and sensor signals of smartwatch devices. In the first stage, to study these relationships, we use a smartphone application to record smartwatch sensor data and the duration of activities with labels provided by the students. Then, collected data are processed with machine learning techniques to classify activities to characterize student behavior.

The paper is organized as follows: in Section 2 we present the methodology for the concept proof, the use of smartwatches and smartphones, and the data mining process for the data. In Section 3 we present results of the experiments, in Section 4 a discussion of the results, and finally in Section 5 the conclusions and future work.

## 2. Materials and Methods

We aim to contribute with technological support for education based on the next hypotheses:

**Hypothesis** **1.** *The variables that affect the academic performance of students are manifested in sensor signals of smartwatches.*

**Hypothesis** **2.** *There is a relationship between signals of smartwatches and daily-living activities of students.*

**Hypothesis** **3.** *It is possible to describe the daily-living activity habits of students with machine learning techniques.*

**Hypothesis** **4.** *There is a relationship between habits of daily-living activity and the academic performance of students.*

**Hypothesis** **5.** *It is possible to provide smart assistance of activity scheduling to achieve a “healthy academic style” that increases the academic performance of students.*

Hypotheses 4 and 5 are only presented to contextualize the current advances in our research. Now, we will be focused on Hypotheses 1 to 3 as guidelines for our concept proof.

**Figure 1:**
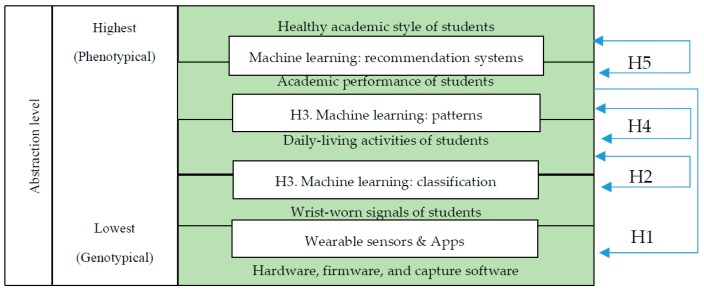
Hypotheses and relationships between smartwatch signals, daily activities, academic performance, and healthy academic style.

Hypothesis 1 is a fundamental starting point where hardware and software are considered to capture wrist-worn signals from students conceptualized at the lowest (genotypical) level of abstraction. Hypothesis 2 links smartwatch signals with daily-living activities at a higher abstraction level (phenotypical) supported by machine learning techniques considered in Hypothesis 3. Figure 1 illustrates these abstraction levels, the hypotheses (abbreviated as H1, H2, H3, H4, H5) and their relationships.

For the concept proof, Computing Engineering undergraduate students were included given their interest, their readiness, and their knowledge of the configuration of smartwatches, smartphones, Android applications, and protocols for uploading data to servers.

**Figure 2:**
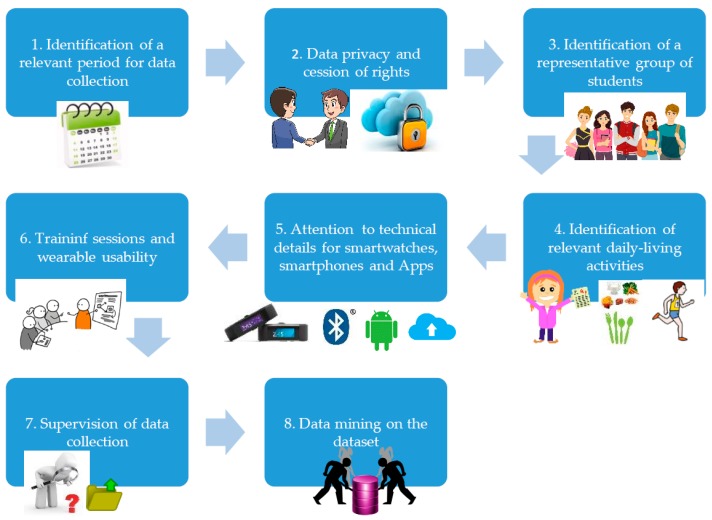
Concept proof methodology for activity monitoring of students with Microsoft Bands, smartphones, and Android Applications.

The concept proof methodology has the following steps, and they are illustrated in Figure 2:

1. Identification of a relevant period in the university calendar for data collection
2. Data privacy and cession of rights
3. Identification of a representative group of students
4. Identification of relevant daily-living activities
5. Attention to technical details for smartwatches, smartphones, and Android applications
6. Training sessions and wearable usability
7. Supervision of data collection
8. Data mining on the dataset

Now, we describe each step of the concept proof methodology.

### 2.1. Identification of a Relevant Period in the University Calendar for Data Collection

The interval for data collection was 8 weeks, from the beginning of the semesterly course to the second examination period approximately, then there are no vacation periods. The students did not share, interchange or relay the smartwatches along the data collection period.

### 2.2. Data Privacy and Cession of Rights

Since this study involves human participants and in accordance with the Federal law of Protection of Personal Data in Possession of Particulars, we obtained an informed consent from each participant. The volunteer participants consented to the processing and generation of statistical reports from their data under conditions of non-disclosure of their explicit identity. Moreover, the participants chose an alias to protect their identity.

The database of this experiment is freely available for download via an institutional server by sending a request to the corresponding author.

### 2.3. Identification of a Representative Group of Students

The group of students included male and female participants. The number of participants was limited to 8 because of the maximum number of available smartwatches. For convenience, the participants were students of Computing Engineering with knowledge on wearable technologies and network communication protocols.

The student sample represented 44% of a total of 18 ascribed to the same group of the latest undergraduate semester of Computer Engineering, so all these students knew each other and shared the same academic scheduling (which was useful for subsequent validation of activity labeling). It should be considered that not all candidate students could be chosen from the full group because some of their smartphones were not compatible with the Android application. There was no monetary stimulus or different treatment with respect to the students who could not or wanted to participate. As an empirical rule, the minimum number of smartwatches were purchased equivalent to almost 50% of the students of the full group. When considering for future experiments, it would be possible to cover almost 100% by using these smartwatches with the other 50%.

As additional information, only one student had a job, so that almost all students were dedicated full time to their university studies.

There was no preference over the age of the participants, except to consider that last semester’s students (about 23 years old) might have more technical experience and might be more responsible when taking care of smartwatches.

### 2.4. Identification of Relevant Daily-Living Activities

Given the importance of the activities, they were carefully chosen to represent, in the best way, the daily habits of the students once we aim to set the academic performance as a function of daily activities, and daily activities as a function of smartwatch sensor signals.

Hence, we applied a survey to get a list of daily-living activities, and a few of them were selected. For example, pet care was initially proposed, but it was discarded given its low voting. Some others were included, such as cinema, but at the end of the experiment, there were no cinema records. We remark that these activities were essentially proposed by the students who were encouraged to keep their habits as natural as possible and no specific scheduling or places were induced for the activities.

### 2.5. Attention to Technical Details for Smartwatches, Smartphones, and the Android Applications

A few technical sessions were required to explain the configuration details of smartwatches, smartphones, and our Android application (App).

#### 2.5.1. Smartwatches

Microsoft Bands smartwatches (*MSBands*) were chosen for the concept proof with the next considerations:

- The availability of a Software Development Kit (SDK) for developing Android applications [25,26];
- The availability of an official application MSBand App with the drivers required to activate a Bluetooth communication between Android smartphones and the Microsoft Bands [27];
- The availability of getting raw data from the *MSBands* sensors;
- The option to configure the *MSBands* sensors via Bluetooth which allows to control their duty cycle (on-off periods) and to get raw data along 24 h.

#### 2.5.2. Smartphones

To enable the Bluetooth communication between a smartphone and an *MSBand* it is necessary to install the MSBand App. The smartphones were the property of the students who felt more comfortable using their own instead of having an additional one.

#### 2.5.3. Data Server

The data server concentrates the uploaded data coming from the App in separated files. The application includes a button to send data files over the Internet to the server. Each filename is unique and considers the student alias and the timestamp. The uploaded files were revised daily to warrant that each student did not forget to upload their data and in such case, an email was sent to the smartphone with the corresponding Microsoft account.

#### 2.5.4. Android Application

Students received instructions on the use of our App that has several configuration options. The alias was captured the first time during the App installation.

**Figure 3:**
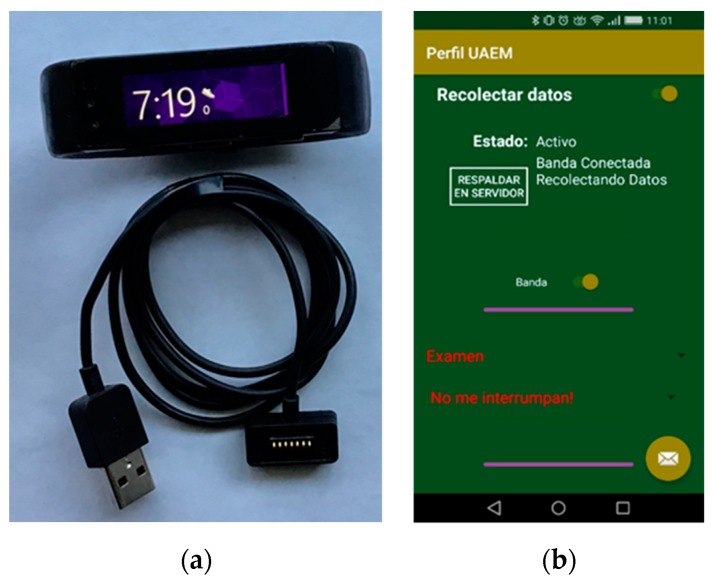
(**a**) Microsoft Band with screen guard protector and USB charger. (**b**) Screenshot of our Android App for data collection in the Spanish version.

Figure 3a illustrates a Microsoft band smartwatch with screen guard protector and USB power charger cable. Figure 3b shows a screenshot of our App with Spanish captions, a start button (“Recolectar datos”), a status indicator (”Activo”), an upload button to send data to the server (“RESPALDAR EN SERVIDOR”), a plain message to know the data collection status (“Banda Conectada Recolectando Datos”), a button to start/stop the *MSBand* sensors (“Banda”), a list-box to choose the current activity (“Examen”), and a list-box to choose the interval of an alert that aims to remember the activity labeling or to disable it in case of sleeping or exam (“No me interrumpan”).

### 2.6. Training Sessions and Wearable Usability

*Smartwatch adjustment and usability*. It was required to verify the wrist adjustment for different students given the three sizes of *MSBands*: small, medium, and large. In the beginning, the use of *MSBands* can lead to stress. It is very important to mention that students were instructed to not stop using the smartwatch and to keep their habits as natural as possible.

There was a learning curve for the proper use of the *MSBands* and the App. The students should have been familiar with them in a few days. There were training sessions about the proper use of the devices. The need for assistance was more notorious at the beginning of the experiment.

### 2.7. Supervision of the Data Collection

Additional to the training session, it was convenient to check the uploaded data to the server to warrant their quality and to detect situations where students did not use the devices properly or when they forgot to label their activities. Those situations were more notorious at the beginning of the experiment as part of the learning curve when using the *MSBand* and our Android App.

As part of a plan to help students not to forget to label their activities and upload the data, we adopted the next actions:

- To verify the uploaded data in the server at the end of the day;
- If a student did not upload data at the end of the day, an email was sent to their smartphone;
- To include alerts in the Android App to remember the activity labeling (it could be activated for half an hour, one hour, two hours or deactivated);
- If a student did not load data for a while, a new student takes his/her place (which only happened once at the beginning of the experiment).

### 2.8. Data Mining on the Dataset

For data mining, we present a second methodology based on the one described in [28] with the next steps:

- Data acquisition;
- Data preprocessing;
- Data segmentation;
- Feature extraction and selection;
- Activity classification.

Now, we describe each one of these steps.

#### 2.8.1. Data Acquisition

The wrist-worn data were acquired with *MSBand* sensors and transmitted to the App, and from the App to the data server for offline processing. Each file size was about 5 MB per day. Each file contains row data with a timestamp, data sensors values, and current activity labeled by the student. The files were organized in folders per day (name format ddmonthyyyy).

The sensor’s data acquired from the *MSBand* correspond to accelerometer, gyroscope, distance, heart rate, pedometer, skin temperature, and calories. Further details of these sensors can be found in the official documentation of the Microsoft Band [25,27].

*MSBand* sensors were activated with a period of 40 s, and a duty cycle of 12.5% for heart rate and 5% for other sensors. It provided up to 24 h of data collection with no battery recharge.

#### 2.8.2. Data Preprocessing

Since each sensor has a different sampling rate, the files contain more rows for faster sensors. To consolidate these data, we latched the latest values of slow sensors to match the rate of the fastest sensor: the gyroscope. Then, new files were generated with a column for each sensor. A subfolder was created for each user and activity (path format ddmonthyyyy\student\activity).

Data cleaning was carefully undertaken to repair or eliminate invalid registers. For example, for a classroom session of 90 min, a student could not finish the activity labeling, and the file could not report more than 90 min. In this case, the data were trimmed after 90 min. To support this decision, classroom scheduling was used to validate the labeled activities.

Further, when data preprocessing tasks were implemented in the App, the data file size was reduced from megabytes to kilobytes. Then, each datafile contained a row for each window-time of 5 min with consolidated values.

#### 2.8.3. Data Segmentation

The files were organized into subfolders named “day-student-activity”, and they were processed to create a single large file where the lasts two columns correspond to activity and student-alias. Additionally, files per activity were created to facilitate data processing.

Afterwards, when data consolidation tasks were introduced in the App, a minimum number of files were required (previously, it was necessary to store files and directories per day, student, and activity). So, data could be stored for several days without compromising the smartphone space, and it also reduced the time required to send the data to the server.

#### 2.8.4. Feature Extraction and Selection

The columns in the datafile correspond to the features that will be used in data classification.

Unixtime was chosen as a timestamp to facilitate data processing, for example, to calculate the average duration of activities.

Some sensors of the *MSBand* provide several measures, for example, the gyroscope delivers three measures x, y, z and the distance sensor provides pace, speed, and total distance.

We distinguished two kinds of measures: instantaneous and differential. Instantaneous measures do not require additional calculation, and they are features identified as follows:

- accelX, accelY, accelZ. Accelerometer measures in *x*-, *y*-, *z*-axis;
- accelXYZ. The sum of the squares of the accelerometer in *x*-, *y*-, *z*-axis;
- distSpeed. The current speed of the band;
- distPace. The current pace of the band;
- gyroXAccel, gyroYAccel, gyroZAccel. The angular velocities in *x*-, *y*-, *z*-axis;
- gyroXYZAccel. The sum of the squares of the gyroscope in *x*-, *y*-, *z*-axis;
- gyroXAnguVel, gyroYAnguVel, gyroZAnguVel. The angular velocities around *x*-, *y*-, *z*-axis;
- gyroXYZAnguVel. The sum of the squares of the angular velocity around the *x*-, *y*-, *z*-axis;
- hrRate. The current heart rate;
- tempSkin. The current skin temperature of the user.

The accelXYZ, gyroXYZAccel, and gyroXYZAnguVel measures were computed within our App. Differential measures need the starting and ending time of the activity, which is obtained using the timestamps:

- pedTotalSteps: The total number of steps;
- cal: The total number of calories;
- distTotal. The total distance.

Data consolidation was developed in window-times of 5 s, and each row was fetched to calculate: (i) average values for instantaneous measures and (ii) absolute values for differential measures. Consolidated data were stored in a (comma-separated values) CSV file with columns (features) listed in Table 1.

At this point, we introduce the wavelet transform that has emerged as an analysis technique where wavelet functions provide time-frequency information of a signal [29]. When analyzing non-stationary signals, wavelet basis provides better results with respect to other basis, such as sine and cosine (used in Fourier analysis), that are well-localized in frequency but oscillate along all the real axis, and then they extract only the frequential representation of a signal. Figuratively, we can say that “Fourier transform gets the musical notes that conforms the music, whereas wavelet transform gets the pentagram” [30].

The discrete wavelet transform (DWT) can be implemented using orthogonal filters [31], and it allows to process digital signals stored in floating point array data, as is the case for the smartwatch sensors data. For the DWT we did not use external libraries, but a Java implementation based on Algorithm 1, where LEVELS define the number of decomposition levels, and h and g are low-pass and high-pass parametric wavelet filters [32,33], respectively.

**Algorithm 1:** Discrete wavelet transform DWT for time-domain feature extraction

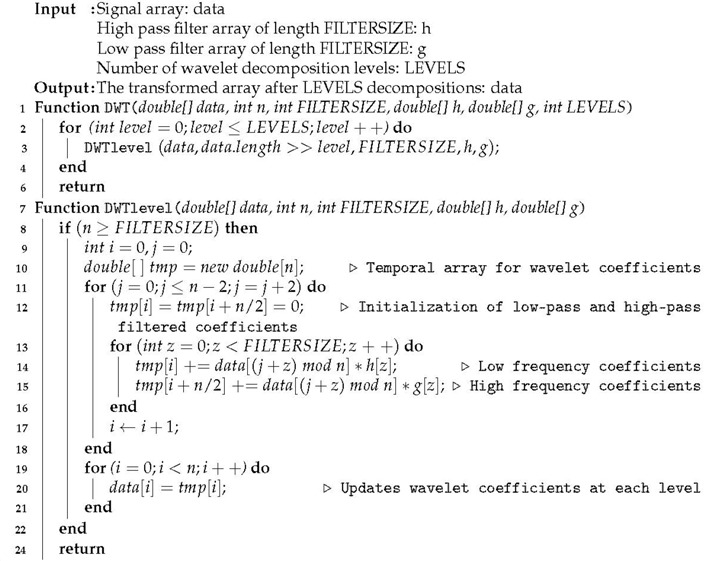



In the experiment, we applied DWT to data buffers of length 128, LEVELS = 4 and *Daubechies-4* filter (FILTERSIZE = 4, parameter 5π/12). Discrete wavelet transform has linear complexity [31], and its implementation did not compromise the battery and performance of the smartphone.

In this way, additional time-frequency features where extracted from accelerometer and gyroscope signals with the DWT by considering four decomposition bands (0, 1, 2, and 3) for signals accelX, accelY, accelZ, gyroXAccel, gyroYAccel, gyroZAccel, gyroXAnguVel, gyroYAnguVel, and gyroZAnguVel. It produced 36 additional features listed in Table 2, where 0-bands are low-pass wavelet coefficient energies, and 1, 2, and 3-bands correspond to high-pass bands energies. The highest frequencies are in the 3-bands.

Finally, the activity labeling provided by the student was included in the CSV file. The activities are eating, running, sleeping, classroom-session, exam, job, homework, transportation, watching TV-Series, and reading.

For attribute selection, we applied the *weka.filters.supervised.attribute.AttributeSelection* filter algorithm is available in the Waikato Environment for Knowledge Analysis software called WEKA [34] with default parameters in three cases: *Case 1. Time domain* uses the features of Table 1; *Case 2. Wavelet domain* replaces accelerometer and gyroscope signals by time-frequency features listed in Table 2; *Case 3. Time and Wavelet domain* combines the features of Cases 1 and 2.

The results of feature selection are identified with “*+selection*” and features extracted with the DWT are referred to as *wavelet domain*:

*Case 1. Time domain+selection.* From 19 features, 11 were selected: accelY, cal, hrRate, pedTotalSteps, tempSkin, distSpeed, gyroXaccel, gyroXAnguVel, gyroYAnguVel, gyroZAnguVel, and gyroXYZAnguVel.

*Case 2. Wavelet domain+selection.* From 43 features (7 from Table 1 and 36 from Table 2) 12 were selected: cal, hrRate, pedTotalSteps, tempSkin, DWTaccelY0, DWTaccelZ0, DWTgyroXAccel0, DWTgyroYAccel0, DWTgyroZAccel0, DWTgyroXAnguVel0, DWTgyroYAnguVel0, and DWTgyroZAnguVel0.

*Case 3. Time and Wavelet domain+selection.* From 55 features (from Table 1 and Table 2) 13 were selected: accelY, cal, hrRate, pedTotalSteps, tempSkin, DWTaccelY0, DWTaccelZ0, DWTgyroXAccel0, DWTgyroYAccel0, DWTgyroZAccel0, DWTgyroXAnguVel0, DWTgyroYAnguVel0, and DWTgyroZAnguVel0.

Note that the three cases include the features cal, hrRate, pedTotalSteps, and tempSkin. The feature selection for Case 2 (12 features) and Case 3 (13 features) differs in a single feature: accelY. Furthermore, note that selected features in wavelet domain correspond only to low-frequency bands.

#### 2.8.5. Activity Classification

We set activity depending on the other features through supervised classification algorithms. The results of classification are shown in Section 3, as well as some other statistical information of collected data.

For data classification we used the next algorithms: MLP [35], Naïve Bayes [36], J48 [37], Random Forest [38]; and JRIP [39]. Cross-validation with 10 folds was used, that means to use 90% of the dataset as training set and 10% as the test set. The results were calculated 10 times by alternating the training and test sets and then calculating the average.

We aimed to get a classification as reliable as possible to create a model for auto-labelling of activities that can be extended to new participants, under the assumption that they will have a similar activity behavior. In this way, we involved the largest possible number of participants by considering the availability of resources in hardware, software, and students time availability.

Additionally, we applied the criterion described in Algorithm 2 to estimate the number of breakfasts, lunches, and dinners based on the timestamp of the instances reported by the participants.

**Algorithm 2:** Estimated number of breakfast, lunches, and dinners**1** If (4 *AM* ≤ *timestamp* & *timestamp* ≤ 9 *AM*) *breakfast***2** If (10 *AM* ≤ *timestamp* & *timestamp* ≤ 5 *PM*) *lunch***3** If (6 *PM* ≤ *timestamp* & *timestamp* ≤ 11 *PM*) *dinner*

## 3. Results

The experiment follows the methodology for the concept proof described in Section 2. The results are 5808 instances of labeled activities distributed as follows: eating 549, running 42, sleeping 3599, classroom-session 90, exam 49, job 243, homework 400, transportation 664, watching TV-Series 147, and reading 25.

Statistical values of the duration of each activity are shown in Table 3. The first column refers to the activity, the second column to the average values, the third column to minimum values, the fourth to maximum values, and the last column to the standard deviation.

To know more about the activities, we estimated the average rate of calories (Table 4) for each activity by considering their duration (see Table 3). In Table 4, the first column refers to the activity, the second to average values, the third to minimum values, the fourth to maximum values, the fifth to standard deviation, the sixth to the average rate (calories/s), and the last to the average rate in calories per hour (calories/h).

Note in Table 4 that the maximum average rate of calories was burned during the exams. In Table 3 the duration of the exams was 4837 s (80 min) which is consistent with the 90 min of the official classroom-session scheduling.

From Table 3 and Table 4, we note that although the sleeping activity hasd an average of 355.3 calories (the closest to the exam). It also has the highest duration of 20,925.5 s (5.8 h), so the rate is 0.02 calories per second (61.1 calories/h). The average calories do not reflect the fluctuations along the activities, so, for example, we cannot deduce the sleep phases and quality, but we can argue that students do not comply the recommendation of sleeping 8 h. For running, the average rate is 0.03 calories per second (51.5 calories per 30 min). Consider, that a goal of our experiment was to collect data along all day without requiring battery recharging, and duty cycle of *MSBand* sensors was adjusted for this purpose, but the price to be paid was a reduction in the measurement accuracy. Moreover, the *MSBand* documentation [25] and research from Stanford University and the Swedish School of Sport and Health Sciences argued that sensors have technical and measurement limitations in comparison with specialized devices used in laboratories [40].

In Table 5 we show statistical values for pedometer distance and speed. Note that except for reading, the sleeping activity has the lowest average distance value (30.1 m), whereas running has the largest average distance value (1488.2 m) with an average speed of 0.6 m/s (~2.2 km/h).

In Table 3 the average duration for running is about 55 min (3303.1 s) with a standard deviation of 35 min (2093.7 s) tied to the duration between 2 min and 2 h (from 126 to 7794 s). In Table 5, running distances vary from 102.4 to 3993.4 m with speeds from 0 to 1.2 m/s (4.3 km/h). Obviously, a zero speed does not correspond to a running status, but it should be considered that students, in their daily scenarios, were not running at constant speed or during fixed intervals (as is reported in other experiments, such as that of Harvard Medical School [41]). Moreover, our App did not include a detailed classification of activities, such as walking or running at several speeds, and the running label was chosen by students as the best choice in the App.

This information, and rules provided by J48 and JRIP, can be used to infer the habits of students and to detect anomalies with the support of experts in disciplines, such as food, sleep, and sport, to provide recommendations that help to enhance the global academic score.

The heart rate sensor provides relevant information for activities. Table 6 shows the average values for heart rate in beats per minute (bpm or beats/min) for each activity.

Note that the lowest average value is for sleeping (69.7 bpm) and the same occurs with its minimum value that reaches 56.4 bpm.

In addition, note in Table 6 that the minimum value for classroom-session is 68.3 bpm which is comparable to the average value for sleeping (69.7 bpm). For exams, the minimum heart rate is 71.7 bpm whereas the maximum heart rate value is 91.9 bpm.

For running, the minimum heart rate is 65.4 bpm (just over sleeping), and the maximum is 91.7 bmp. It may be that the “running” label is not the best one after reviewing the results of our experiments, but in spite of this label, it is important to mention that *MSBand* documentation for heart rate sensor prevents its use only in resting mode, since the SDK does not provide access to the heart rate values optimized for any other activity.

Note that heart rate values for running are at a typical heart rate zones for running [42] calculated by subtracting the age from 220 (Max Heart Rate MHR = 220 − 23) and a moderate intensity zone is 50% to 70% (98.5 to 147.8 bpm), so we consider that students followed a more passive behavior than typical running, and it suggests the need for consulting experts in this field to detect abnormalities in dedication and effort for physical activities.

The results of applying Algorithm 2 to the dataset that contains 59 feeding instances reported by the students were: 23 breakfasts, 17 lunches, and 19 dinners. Since 59/3 = 19.7, and by considering the duration from Table 3, we argue that students follow a balanced number of meals per day with an average duration of 33 min.

As we mentioned in Section 2, for data classification we used MLP, Naïve Bayes, J48, Random Forest, and JRIP classifiers. MLP was the slowest algorithm with default parameters of WEKA. Naïve Bayes was fast but achieved the worst accuracy. In spite of the fact that MLP achieved a competitive accuracy compared with J48 and JRIP, we prefer rule-based models over the network model of MLP.

So, we chose J48, JRIP, and Random Forest by considering their performance, classification accuracy, and interpretability.

We consider two main factors for misclassification: the former due to human error when labeling the activity in the App, and the second due to the classification capacity of the algorithm. The first, was treated in the data preprocessing step described in Section 2. The last was treated by testing several supervised algorithms and choosing the best one.

Table 7 shows the first column with six cases of the study described in Section 2. The number of input features are shown in the second column, and the classification accuracy in the third, fourth, fifth, sixth, and seventh columns for MLP, Naïve Bayes, J48, Random Forest, and JRIP classifiers, respectively.

In Table 7, the worst cases (64.9% and 73.2%) are for Case 1 with 11 features of Naïve Bayes and JRIP. The highest accuracy is 87.2% for Case 3 with 55 features and Random Forest, and 86.9% with 13 features.

Although Random Forest achieves good accuracies, it is slower and more complex than J48 and JRIP. Moreover, J48 and JRIP are relevant for our purposes since they produce rules that can be used within the App where battery and hardware resources are limited.

For J48, the best performance is 83.3% for Case 3 with 13 features. This result outperforms Case 1, and it reflects the importance of using wavelet features.

JRIP has a similar performance to J48 and is a rule-based algorithm, that facilitates the interpretability of the classification.

**Figure 4:**
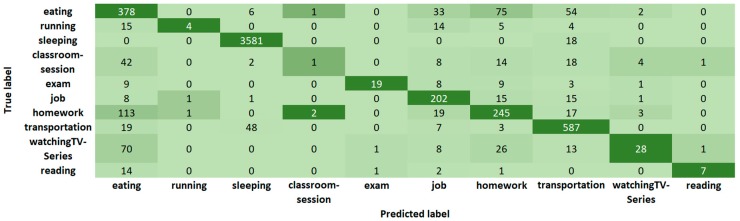
Confusion matrix for daily-activity classification with Random Forest.

**Figure 5:**
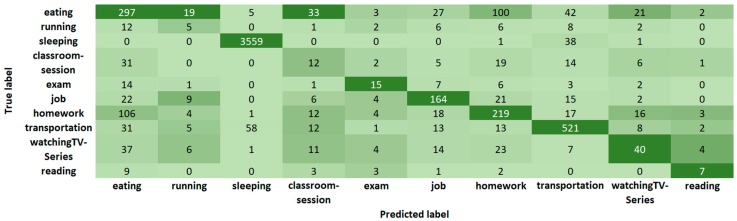
Confusion matrix for daily-activity classification with J48.

**Figure 6:**
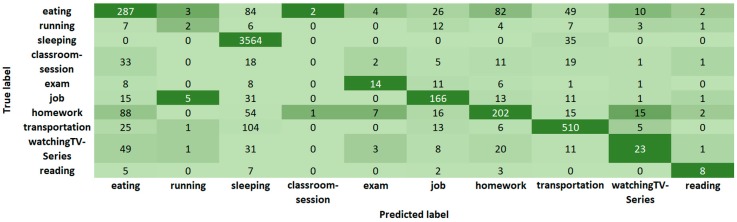
Confusion matrix for daily-activity classification with JRIP.

In Figure 4, we show the confusion matrix of Random Forest for Case 3 with 13 features and ten activities. Figure 5 shows the confusion matrix of J48 and Figure 6 the confusion matrix of JRIP, also for Case 3 with 13 features and ten activities. The ten columns represent the output (Predicted label) of the classification algorithm compared with the first column that corresponds to the (True) label given by the students. Note that we have a non-balanced number of activities because of the duration of the activity, its frequency, and the number of labeled activities. For example, in Table 3 we show that the average sleeping time is 20,925.5 s (5.8 h) while the average time for running is 3303.1 s (0.9 h) that yields to have more sleeping instances than running instances. Moreover, consider that these instances correspond to window-time of 5 min.

## 4. Discussion

Hypothesis 1 is fundamental for this research since it establishes a relationship between smartwatch sensors signals and academic performance.

Experimental results of Section 3 support the relationship between smartwatch signals and daily-living activities of students (Hypothesis 2) by achieving a classification accuracy of 87.2%.

It is very important to remark that participants were encouraged to carry out their daily activities as naturally as possible. So, no specific scheduling or places were induced for the activities, and smartwatches and smartphones were configured for monitoring along all day. The classification is supervised since students provided activity labeling.

Although there are several works related to daily activities, they often comment about the need for exploring the activities in more realistic scenarios. So, this is an important difference with our experiment.

To support Hypothesis 3, five classifiers were tested: MLP, Naïve Bayes, Random Forest, J48, and JRIP. MLP was the slowest algorithm, and Naïve Bayes provided the worst classification accuracy, so they were discarded. Random Forest outperforms J48 and JRIP, although it is slower and more complex. J48 and JRIP offer a competitive classification accuracy, and their models are easier to interpret and to implement in a smartphone application or embedded software.

The feature extraction and selection we developed highlight the importance of measuring calories, heart rate, pedometer distance, and skin temperature, together with gyroscope and accelerometer signals. In addition, it is noticeable from Table 7 that wavelet features increase the accuracy.

JRIP built 39 rules to model the activities. For example, Algorithm 3 shows rules obtained with JRIP to characterize exam and reading activities. The complete model is available upon request.

**Algorithm 3:** Exam and reading rules from JRIP

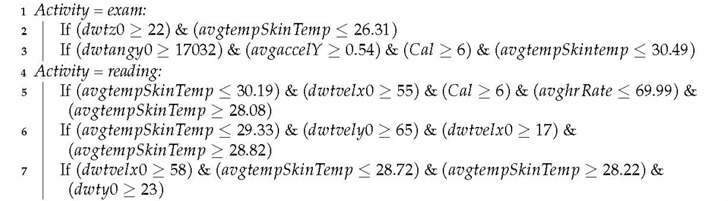



Statistical analysis of collected data gives us information about activities. For example, from Table 3 to Table 6 we note that:

- Students spent about one hour (3918 s ~ 1 h) in transportation.
- Students do not comply with the recommendation of sleeping 8 h (average 5.8 h).
- Students have three meals per day (breakfast, lunch, and dinner) with an average duration of 33 min.
- Students burn the maximum calories per second (0.12 calories/s) during an exam, almost six times more than running.
- Students burn an average of 103 calories per hour in activities labeled as “running” with variations from 2 min to 2 h, and speed from 0 to 1.2 m/s (4.3 km/h) that suggests the inclusion of more activities in the App to pay special attention in their dedication and effort in sport activities, once these values are out of the range 240 to 355 reported in [41] for the slowest speed of running (5 mph, 8 km/h); and to deal with mixed activities presented in more realistic scenarios, such as watching TV and eating, walking and drinking water, since a crisp classification displayed in list-boxes seems to be insufficient, but a fuzzy selector could be an alternative.
- Some students reach an average sleeping heart rate of 68.3 bpm in a classroom-session which is close to the average for sleeping (69.7 bpm). We confront this with confusion matrices of Figure 4, Figure 5 and Figure 6, where classroom-sessions are confused with other activities including sleeping.

The similarity of heart rate values makes us think about the possibility of identifying unwanted or abnormal situations during activities, such as classroom-sessions: Are students falling asleep in the classroom? Rules obtained with algorithms, such as J48 and JRIP, could help us to understand and detect these situations and in the future (supported by experts and after outperformed our dataset) to obtain enough validity to generate smartwatch vibration alarms or to build a dashboard for the teacher where abnormal or undesirable situations are displayed.

Despite *MSBands* being out of stock, and its SDK no longer available, some other devices can be used or implemented guided by this research by considering wavelet feature extraction as part of the smartphone applications or embedded systems.

If habits of daily-living activities determine the academic performance (Hypothesis 4), then we get a transitive dependence of smartwatch signals and academic performance. At this stage of our research, the results open the possibility of developing future experiments with automatic activity-labeling [43] that facilitates the recognition of activity patterns to propose a recommendation system that helps to enhance academic performance, which is the magnificent Hypothesis 5.

Finally, we comment that the implementation of data mining task inside smartphone applications make it useful in applications where real-time decision is required, such as safe driving or assistance for a medical emergency.

## 5. Conclusions

We present a methodology for a concept proof to evaluate the recognition of daily-living activities of students using smartphone sensors and an Android App configured as master-slave for monitoring along all day.

Several hypotheses were considered concerning the relationship of smartwatch sensor signals, daily activities, and academic performance.

J48, JRIP, and Random Forest algorithms were evaluated in recognizing daily-activities with acceptable results by considering that no specific scheduling or places were induced for the activities and that students provided the labeling of their own activities.

The classification model for activities opens the possibility of developing future experiments with automatic activity labelling that also allows to improve our dataset.

Statistical analysis and rule extraction from collected data give us information about the students’ behavior, and rule-based JRIP algorithm provides easy interpretability from low-level sensor data to a higher abstraction level of human activities.

Current results support three of our five hypotheses, and it remains as future work to study the relationship with academic performance through the identification of patterns from good and bad habits of students to propose recommendation systems that yield to a healthy academic style.

## Figures and Tables

**Table 1:** Features for data classification.

Number of Features	Sensor/Source	Feature
1	timestamp	unixtime
4	accelerometer	accelX, accelY, accelZ, accelXYZ
3	distance	distSpeed, distPace, distTotal
8	gyroscope	gyroXAccel, gyroYAccel, gyroZAccel, gyroXYZAccel, gyroXAnguVel, gyroYAnguVel, gyroZAnguVel, gyroXYZAnguVel
1	heart rate	hrRate
1	temperature	tempSkin
1	pedometer	pedTotalSteps
1	calories	cal

**Table 2:** Wavelet features for data classification.

Number of Features	Sensor/Source	Feature
12	accelerometer	DWTaccelX0, DWTaccelX1, DWTaccelX2, DWTaccelX3 DWTaccelY0, DWTaccelY1, DWTaccelY2, DWTaccelY3 DWTaccelZ0, DWTaccelZ1, DWTaccelZ2, DWTaccelZ3
24	Gyroscope	DWTgyroXAccel0, DWTgyroXAccel1, DWTgyroXAccel2, DWTgyroXAccel3, DWTgyroYAccel0, DWTgyroYAccel1, DWTgyroYAccel2, DWTgyroYAccel3, DWTgyroZAccel0, DWTgyroZAccel1, DWTgyroZAccel2, DWTgyroZAccel3, DWTgyroXAnguVel0, DWTgyroXAnguVel1, DWTgyroXAnguVel2, DWTgyroXAnguVel3, DWTgyroYAnguVel0, DWTgyroYAnguVel1, DWTgyroYAnguVel2, DWTgyroYAnguVel3, DWTgyroZAnguVel0, DWTgyroZAnguVel1, DWTgyroZAnguVel2, DWTgyroZAnguVel3

**Table 3:** Activity duration in second: average, minimum, maximum and standard deviation.

Activity	Avg (s)	Min (s)	Max (s)	StDev (s)
eating	1995.7	147	5134	1218.3
running	**3303.1**	126	7794	2093.7
sleeping	**20,925.5**	345	33,076	7083.4
classroom-session	5808.1	3036	9613	1474.0
exam	**4837.0**	1641	7156	1774.4
job	13,713.0	1501	45,619	15,066.4
homework	6743.5	513	18,338	4807.5
transportation	3918.1	310	11,716	2666.3
watching TV-Series	4934.3	604	16,124	4692.5
reading	7364.0	7364	7364	0

**Table 4:** Calories by activity.

Activity	Calories				Calories/s	Calories/h
	Average	Min	Max	StDev		
eating	41.7	2	134	27.0	0.02	75.2
running	94.5	2	274	68.0	0.03	103
sleeping	**355.3**	9	721	148.3	**0.02**	**61.1**
classroom-session	109.2	61	228	39.5	0.02	67.7
exam	595.3	27	6094	1658.6	**0.12**	**443**
job	193.9	19	618	212.6	0.01	50.9
homework	138.9	12	351	98.7	0.02	74.2
transportation	83.5	7	311	58.3	0.02	76.7
watching TV-Series	102.9	9	357	109.2	0.02	75.1
reading	171.0	171	171	0	0.02	83.6

**Table 5:** Distance by activity.

Activity	Distance							
	Total Distance (m)				Speed (m/s)			
	Average	Min	Max	StDev	Average	Min	Max.	StDev
eating	64.8	0	861.0	108.3	0	0	0.2	0
running	**1488.2**	102.4	3993.4	827.1	**0.6**	0	**1.2**	0.4
sleeping	**30.1**	0	295.2	61.9	0	0	0.1	0
classroom-session	85.8	0	306.5	83.1	0	0	0.1	0
exam	129.8	0	804.9	224.8	0	0	0	0
job	1123.5	0	2615.6	995.5	0.1	0	0.6	0.2
homework	157.4	0	1195.9	244.6	0	0	0	0
transportation	743.0	0	2840.6	645.5	0.2	0	0.8	0.2
watching TV-Series	146.1	0	1280.2	346.2	0	0	0.2	0
reading	21.0	21.0	21.0	0	0	0	0	0

**Table 6:** Heart Rate by activity.

Activity	Heart Rate (bpm)			
	Average	Min	Max	StDev
eating	76.8	65.8	113.2	6.8
running	76.6	**65.4**	**91.7**	6.0
sleeping	**69.7**	**56.4**	107.2	7.5
classroom-session	76.1	**68.3**	93.1	6.3
exam	76.8	**71.7**	**91.9**	5.5
job	75.0	69.2	85.0	4.9
homework	76.7	68.8	113.3	8.3
transportation	74.8	65.7	94.7	4.7
watching TV-Series	74.3	68.8	79.8	3.1
reading	70.0	70.0	70.0	0.0

**Table 7:** Activity classification accuracy for MLP, Naïve Bayes, J48, Random Forest, and JRIP algorithms.

Features for Cases of Study	Number of Input Features	MLP	Naïve Bayes	J48	Random Forest	JRIP
Case 1. Time domain	19	74.3%	65.9%	76.6%	82.1%	73.9%
Case 1. Time domain + selection	11	73.1%	64.9%	76.1%	81.6%	73.2%
Case 2. Wavelet domain	43	81.6%	74.6%	81.1%	85.1%	80.8%
Case 2. Wavelet domain + selection	12	81.8%	79.8%	81.1%	85.1%	80.3%
Case 3. Time and wavelet domain	55	**83.3%**	75.5%	82.8%	**87.2%**	82.2%
Case 3. Time and wavelet domain + selection	13	83.9%	80.6%	83.3%	**86.9%**	82.2%
